# Continuous and Real-Time Detection of Drinking-Water Pathogens with a Low-Cost Fluorescent Optofluidic Sensor

**DOI:** 10.3390/s18072210

**Published:** 2018-07-10

**Authors:** João Simões, Tao Dong

**Affiliations:** 1 Institute of Applied Micro-Nano Science and Technology (IAMNST), Chongqing Key Laboratory of Colleges and Universities on Micro-Nano Systems Technology and Smart Transducing, Chongqing Engineering Laboratory for Detection, Control and Integrated System, National Research Base of Intelligent Manufacturing Service, Chongqing Technology and Business University, Nan’an District, Chongqing 400067, China; joaonetgomes@gmail.com; 2 Department of Microsystems (IMS), Faculty of Technology, Natural Sciences and Maritime Sciences, University of South-Eastern Norway, Postboks 235, 3603 Kongsberg, Norway

**Keywords:** pathogen detection, drinking water quality, intrinsic fluorescence, tryptophan, real-time detection, continuous monitoring, on-line optofluidic sensor, low-cost instrumentation, optofluidic

## Abstract

Growing access to tap water and consequent expansion of water distribution systems has created numerous challenges to maintaining water quality between the treatment node and final consumer. Despite all efforts to develop sustainable monitoring systems, there is still a lack of low cost, continuous and real time devices that demonstrate potential for large-scale implementation in wide water distribution networks. The following work presents a study of a low-cost, optofluidic sensor, based on Trypthopan Intrinsic Fluorescence. The fluorospectrometry analysis performed (before sensor development) supports the existence of a measurable fluorescence output signal originating from the tryptophan contained within pathogenic bacteria. The sensor was mounted using a rapid prototyping technique (3D printing), and the integrated optical system was achieved with low-cost optical components. The sensor performance was evaluated with spiked laboratory samples containing *E. coli* and *Legionella*, in both continuous and non-continuous flow situations. Results have shown a linear relationship between the signal measured and pathogen concentration, with limits of detection at 1.4 × 10^3^ CFU/mL. The time delay between contamination and detection of the bacteria was practically null. Therefore, this study supports the potential application of tryptophan for monitoring drinking water against water pathogens.

## 1. Introduction

In 2015, 71% of world population has access to a safe drinking water source [1]; this was one of the most important public health improvements achieved globally. Despite this significant improvement, there are still two billion people that use drinking water sources which are contaminated with feces [1]; this clearly demonstrates that, despite the advances, we are still far from achieving worldwide drinking water safety. For this reason, in the same year, the European Commission stated: “There is a strong need for new water monitoring and control systems to reduce unnecessary water analyses and concentrate on the threats that really matter.” [2]. This statement clearly criticizes the current water safety strategy, where most of the efforts have been in the direction of improving the water treatment process, which is the primary means of securing water safety, but by itself does not ensure final consumer safety. This misconception comes from the fact that DWDSs (Drinking Water Distribution Systems) are generally considered “contamination free”. However, through DWDSs, a high number of contamination cases can occur, among which the most common are biofilm deposition [3], pressure losses [4,5], aging of DWDSs, and short periods of poor treatment and rain induced infiltrations. All these possible scenarios are responsible for decreasing the water quality/safety, and are normally associated with pathogenic intrusions. For this reason, water monitoring plans are starting to include DWDSs monitoring as a major step for improving drinking water safety [6].

Traditional water quality control us normally done through the collection of water samples at the end point of the network followed by laboratory analysis, which is often conducted with cell cultures or other time consumption methods [7]. This approach is highly specific to the identification of the contaminant microorganism, but with an increase in the dimension of the DWDSs, it is also becoming more expensive to follow the regulations for this control. Besides the economic future impracticability of this approach [8], the method itself has several flaws. The most common are: (i) that measuring the water quality only at the end point does not locate the source of contamination, (ii) cell culture methods take a significant time to provide accurate results (by the time the contamination is detected, it is possible that thousands of people are already affected [9]), and (iii) the possibility of short time contamination events which are very difficult to detect by methods which do not provide continuous and real time measures.

Due to the inefficiency of current approach, alternatives strategies, such as Internal Networking Monitoring, have started to emerge. The advantage of such approaches comes from the fact that, with a sufficient number of strategically-placed monitoring nodes and a detection method that provide accurate measures in NRT (near real time), it is possible to locate contamination origin and monitor how fast the contamination spreads through the network, allowing remedial measures to be taken immediately [10]. Despite the overwhelming advantages of Internal Networking Monitoring, the actual implementation of this system poses a huge challenge due to the lack of cost-effective methods that can detect pathogens in NRT. Based on this need, recent reported microdevices have tried to replicate traditional laboratory techniques, such as real time PCR in a microchip [11]. But despite advances in NRT detection systems, the instrumentation is still expensive, and although claims of real-time capability have been made, a delay of some minutes between event and detection still occurs. 

As this problem is yet to be solved, alternative techniques that take advantage of biomarkers that are found in bacteria cells are being developed. The best example is the ATP bioluminescence assay, which is based on the use of an ATP marker to detect presence of bacteria. The reaction assay is based on the D-luciferin oxidation by the luciferase, where the emitted light from the bioluminescent reaction is proportional to the ATP concentration [12,13]. This method is considered one of the most sensible and rapid (NRT) for microbial detection; its only downside is the need for expensive reagents. ATP bioluminescence may solve the problem of real-time detection, however, due to the high cost of reagents, it is impracticable for mass distribution on DWDSs, which leaves current water monitoring systems without an optimal solution to meet the overall requirements.

A recent review work [14] that compared spectroscopy techniques has acknowledged fluorescence as a method with “low exposure times, low detection limit, with broad spectral features”. This perfectly matches the requirements for Internal Network Monitoring applications. Based on this, the present work explores a “natural fluorescence technique” called Intrinsic Fluorescence. This method is based on the fluorescence emitted by microorganism when exposed to target UV light. This technique is label free, and still preserves the rapid detection time from traditional fluorescence techniques. Compared to other fluorescence techniques requiring labelling with artificial fluorophores [15], the intrinsic fluorescence technique is less expensive because there is no need for extra reagents. This characteristic makes this method perfect for mass distribution through the network, and since there is no need for reagents, the operation costs are practically null; the only cost is from the sensor production.

This work presents a low-cost optofluidic sensor based on the intrinsic fluorescence technique of the tryptophan biomarker. First spectroscopy analyses were conducted to evaluate the optimal tryptophan fluorescence excitation and emission wavelength for implementing the sensor, and to confirm if the signal was able to distinguish interfering substances present in drinking water. After the selection of the optimal wavelength, a fluidic biosensor was constructed for testing the method in both offline and online situations with two types of bacteria commonly found in contamination events. The magnitude of the signal and delay were characterized with the new sensors. The sensor prototype has exploited a fast prototyping method (3d Printing), and the optoelectronic system developed in accordance with the signal requirements.

### 1.1. Theory

Intrinsic Fluorescence principle has been used for a long time in protein analysis and other molecular studies. This fluorescence light come from molecules denominated as fluorophores, such as tryptophan, tyrosine, phenylalanine, nucleic acids, flavins, cytochromes etc. [16]. For this method, when the UV light reach a fluorophore, it causes it to go into an excitation state and makes it undergo conformational changes (partly dissipating some of this energy); when the fluorophore returns to the normal state, a light at a different wavelength is emitted [17]. For this process to be to measured and quantified, two essential characteristics should be considered. The first is quantum yield; this is represented by the ratio between the photon emitted and absorbed. Only fluorophores with reasonable quantum yields can be measured, otherwise the excitation source would need an ultra-high radiant output to reproduce a measurable signal. The other essential characteristic is the stoke shift, that is defined as the difference between the excitation and emission wavelength. Normally high stoke shifts are desirable because they allow none signal overlapping, which allows us to differentiate between both signals (excitation and emission).

#### 1.1.1. Essential Aminoacid Intrinsic Fluorescence Properties

Amino acids are the basic constituents of all living cells, including pathogenic bacteria. The selected fluorophore to indicate pathogen presence in drinking water was Tryptophan, which is one of the three essential amino acids with Intrinsic Fluorescence properties. This amino acid selection between the three possible options come from the fact that Tryptophan has the highest quantum yield (20%) and stoke shift (70 nm), as we can see in Table 1.

#### 1.1.2. Sensor Detection Principle

**Figure 1:**
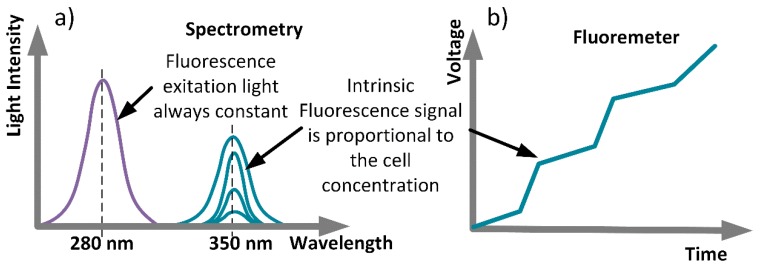
(**a**) Excitation and emission Spectro on a Fluro-spectrometer; (**b**) Signal output from a Fluorometer.

Spectrofluorometry (Figure 1a) is a standard technique used to run full spectrum analysis. The instrumentation is expensive, and the analysis time depends on the spectrum range. By selecting only an excitation wavelength and an optimal emission wavelength, in our case for Tryptophan, it is possible to significantly reduce the instrumentation and size needed for the device. It is also possible to have a completely real-time sensor. For that, a PMT (photomultiplier tube, with an output voltage is used; it is possible to obtain a voltage signal that is proportional to the pathogen concentration in the liquid sample (Figure 1b).

## 2. Materials and Methods

### 2.1. 3D Printing Design and Production

**Figure 2:**
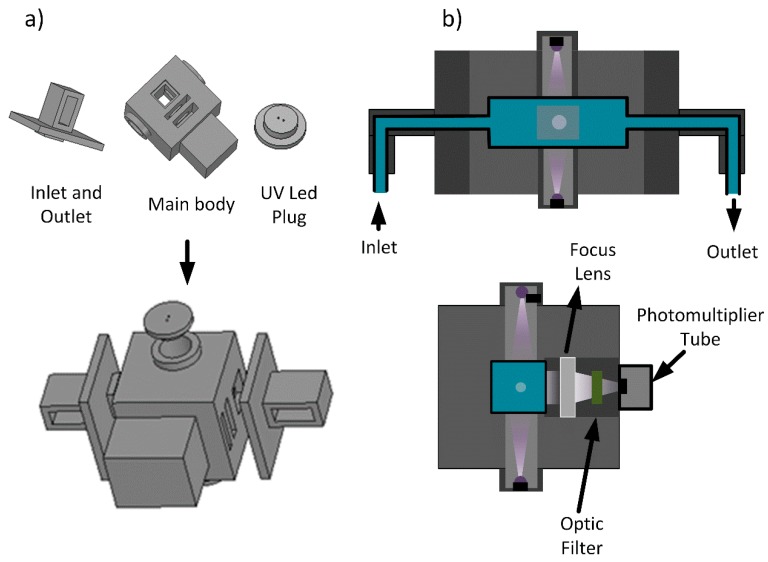
(**a**) Design of the parts for the 3D Printer model; (**b**) Schematic of the system assembly in addition with the optic system, from two different views.

The system design was realized using the *Design Spark Mechanical2.0* (Figure 2a). For an easier assembly of the system, the prototype was divided into small parts that were pressed together for the final device. The model printing was done using Black PLA (Polylactide Acid), heated at 200 °C [21]. Due to the machine resolution, the parts that are assembled together were given a 0.2 mm tolerance so they can be pressed together while maintaining system integrity. 

### 2.2. Optical System

The light source used was two commercial Deep UV Light Emitting Diodes (LEDs), with a 6° view angle, so light dispersion would be minimized until it reached the sample. Both light sources were displaced at 90 °C (Figure 2b) from the detector to optimize the signal to noise ratio [22]. An optic bandpass filter with a 340 nm central wavelength and 20 nm bandwidth, was used to block light that comes from scattering the water molecules, as well as other external interferences. The detector used was a Photomultiplier tube (Hamamatsu H17023). For the optic lens, a new focal distance needed to be calculated for the PMT window to be a size of 10 mm; all calculations are explained in the following subchapter.

#### Focal Distance 

**Figure 3:**
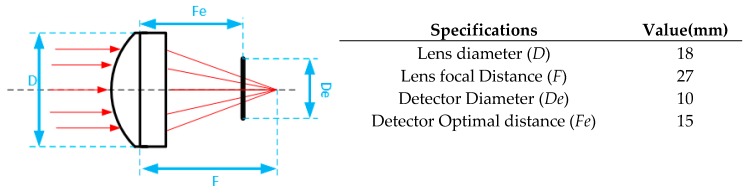
Illustration of the lens schematic and specifications used on calculations.

The Convex Plan lens can be considered an infinite/finite conjugate system [23], where the objective is to optimize the luminance reaching the detector. This system can be described by the following equation:

(1) $$f=\left(\frac{1}{2\times NA}\right)=\left(\frac{F}{D}\right)$$

where *f* is the lens ability to focus light, which has 1.5 value (from manufacture), *NA* is the lens numerical aperture 0.33, *F* is the focal point distance, and *D* (18 mm) is the Lens diameter. Following Equation (1), *F* has a final value of 27 mm. Considering now the detector as a window of 10 mm, *De*, the optimal distance for maximizing the luminance, *Fe*, needs to be less than the Focal distance, because we want to focus the light on an area instead of a single point (Figure 3). This distance can be simple calculated by:

(2) $$\frac{Fe}{De}=\frac{F}{D}$$

The final, optimal distance between the lens and the detector as the value of 15 mm.

### 2.3. Readout System

**Figure 4:**
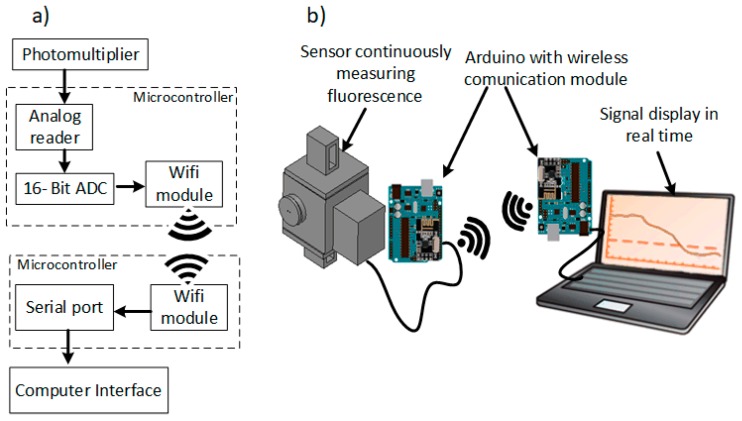
(**a**) Schematic of the signal processing and transmission; (**b**) Components used for the signal processing and transmission.

The PMT outputs an analog signal from 0 V to +5 V, proportional to the light intensity reaching the detection window. For the signal digital conversion to be realized without losing resolution, an external 16-bit ADC (ADS1015) was connected to an Arduino Uno R3 (Figure 4b). After the digital conversion, a 2.4 GHz RF transceiver (NRF24L01 Transceiver, Nordic Semiconductor, Trondheim, Norway) was connected to the microcontroller, and was continuously transmitting data with a 1 Hz sampling frequency (the microcontroller read the PMT signal once every second). A MATLAB interface was also developed in which it is possible to visualize the signal in real-time. A power source was designed on a PCB, which was projected to be connected to a battery. The system requirements are 10 V for the UV LEDs, +5 V, −5 V and 0.5 to 1.1V (gain adjustment) for the Photomultiplier, and +10 V to Arduino Uno R3.

### 2.4. Experimental Procedure 

#### 2.4.1. Instrumentation

For Fluorospectrometry tests, an FS5 Spectrofluorometer (Edinburgh Instruments, Livingston, UK) was used. Therefore, it was possible to realize full spectrum analyses to select the optimal emission and absorption wavelengths for Tryptophan, and to evaluate possible signal interferences on the same wavelength (as Raman scattering).

**Figure 5:**
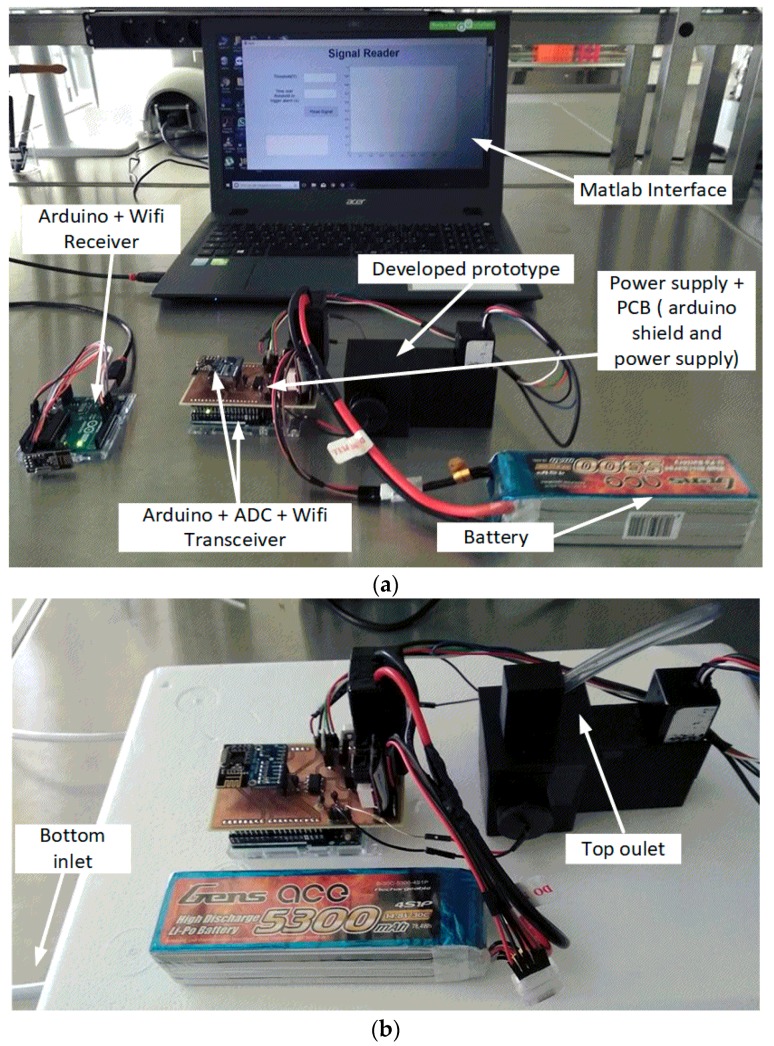
Experimental prototype developed for continuous detecting the bacteria in water. (**a**) Setup used for offline tests; (**b**) Setup used for the online tests.

Both Offline (samples in stationary state), Figure 5a, and Online (water continuously flowing through the device), Figure 5b, tests were performed using the developed step up. The online tests were performed with the assistance of a pump and some fluidic equipment (tubes, isolating material, etc.) to make the water flow continuously through the device. All results were recorded with the developed interface. A flow-through Suprasil quartz cuvette with a 10 × 10 mm pathlength was used as an analysis chamber.

#### 2.4.2. Sample Preparation

The experiment used freeze dried *E. coli* (K-12 strain) and *Legionella Waltersii*, to simulate contaminant pathogens. First, for *E. coli*, a cell culture in standard Luria-Bertani broth (LB) was prepare overnight at 130 rpm and 37 °C [24]. The *Legionella Waltersii* was incubated at 37 °C and 3% CO_2_ on buffered charcoal-yeast extract (BCYE) agar plates for several days, until the quantity of bacteria was sufficient [25]. The samples were then centrifuged at 4000 rpm for 4 min and washed in a PBS (phosphate buffered) solution to remove the growth medium [26]. This step was repeated three times. The bacteria were then mixed in in DI and Tap water.

**Figure 6:**
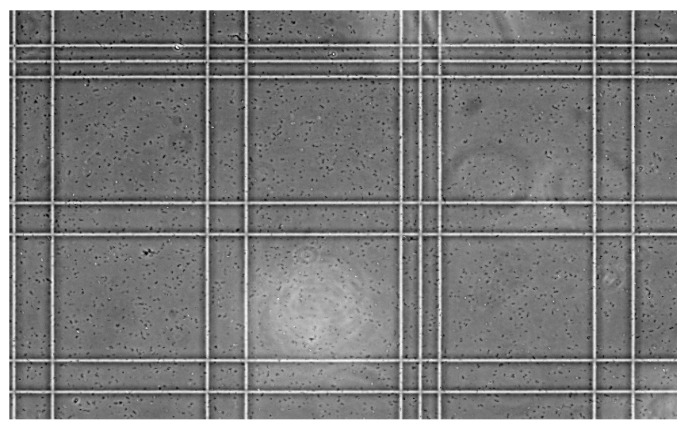
Microscope grayscale image of the cell in the counting chambers used for cell quantification. Each square measure 0.2 mm × 0.2 mm × 0.1 mm.

For the cell count procedure, a Microscope and a standard technique based on counting chambers (Figure 6) [27] were used, where it was possible to determine the number of cells per volume of liquid. The equation used to determine the final number of cells per volume was:

(3) $$Particle\;per\;volume=\frac{Counted\;particles}{Counted\;surface(mm^{2})\times Chamber\;depth(mm)\times Dilution}$$

## 3. Results

### 3.1. FluoroSpectrometry

To verify the optimal Tryptophan Intrinsic Fluorescence wavelength in order to implement it in the sensor, multiple excitation wavelength ranging from 268 nm until 288 nm, with a 2 nm step, were tested. The emission wavelength was measured from 300 nm to 380 nm in all performed scans. 

**Figure 7:**
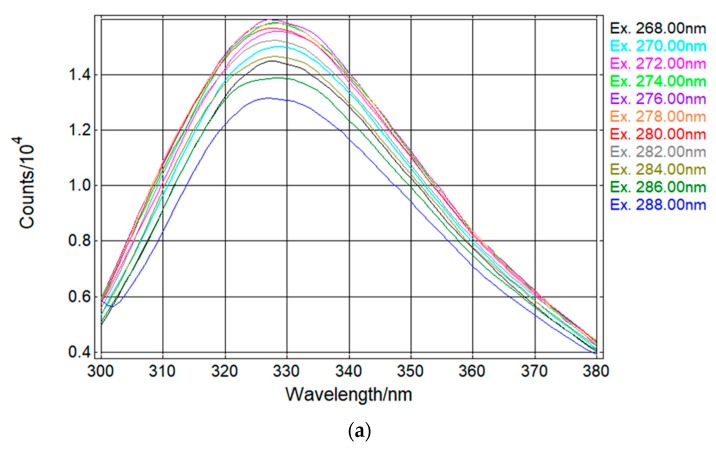

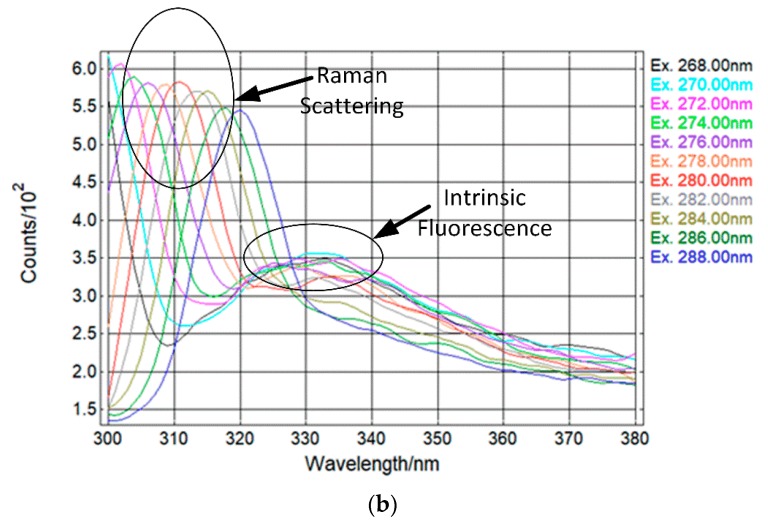
Spectrometry analysis on the Spectrofluorometer with a tap water sample containing high *E. coli* concentration (>1 × 10^6^ CFU/mL) (**a**); and lower *E. coli* concentration (<1 × 10^5^ CFU/mL) (**b**).

In Figure 7a, we can see the Tryptophan signal for a tap water sample spiked with a high concentration of *E. coli* (>1 × 10^6^ CFU/mL). (Regarding the various wavelengths tests, we can see that the signal has a higher emission at wavelengths near 280 nm, and starts decreasing when the wavelength distance increases to 280 nm. In Figure 7b, we have a spiked sample with a lower bacteria concentration (<1 × 10^5^ CFU/mL). An effect called Raman Scattering [28] started to affect the signal; this effect can be nullified in the prototype by placing a filter that will block light wavelengths lower than 315 nm.

### 3.2. Offline Measurements 

The following results present the preliminary tests performed with the developed sensor. In these studies, the objective was to measure the relation between the Tryptophan Intrinsic Fluorescence and the bacteria concentration in tap water and DI (Deionized water). In this experiment, a quartz Suprasil cuvette was placed inside the device (each cuvette takes approximately 3 mL of solution). Before the contaminated samples measurements were taken, negative control tests were performed. The average background noise found was 0.03V for tap water and 0.02 V for DI water.

**Figure 8:**
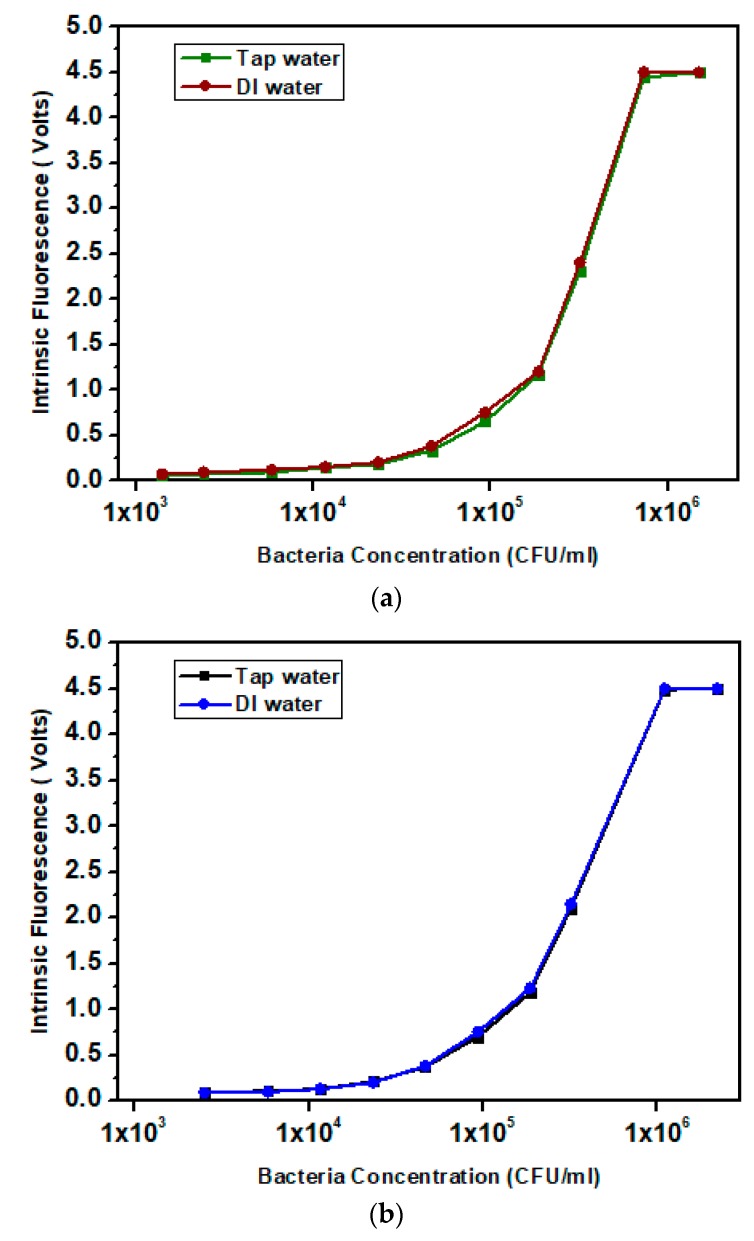
Intrinsic Fluorescence measures on spiked samples with *E. coli* (**a**) and *Legionella* (**b**).

For the tests, a dilution series was prepared, where a starting sample with a concentration of 1.5 × 10^6^ CFU/mL (*E. coli*, Figure 8a) and 2.2 × 10^6^ CFU/mL (*Legionella*, Figure 8b) was used. From that samples, dilutions were performed until the detection limit of the system was reached. The PMT gain was adjusted for each pathogen curve to obtain a higher signal to noise ratio and allow both signals (*E. coli* and *Legionella*) to have a similar output value for the same pathogen concentration

Each test was performed in triplicate and the results presented are the average values of each measurement. In these experiments, both *E. coli* and *Legionella* show a linear relation between intrinsic fluorescence and pathogen concentration. The pathogen concentration scale was expressed in the log_10_ scale of CFU/mL and the Intrinsic Fluorescence signal in linear scale (volts).

On the first two measures, the signal was constant due to the PMT saturation, which put the highest concentration detectable at around 7.5 × 10^5^ CFU/mL for *E. coli* and 1.1 × 10^6^ CFU/mL for *Legionella*. The lower detection limit was approximately 1.4 × 10^3^ CFU/mL for *E. coli* and 2.1 × 10^3^ CFU/mL for *Legionella*. 

#### 3.2.1. Calibration Curve 

**Figure 9:**
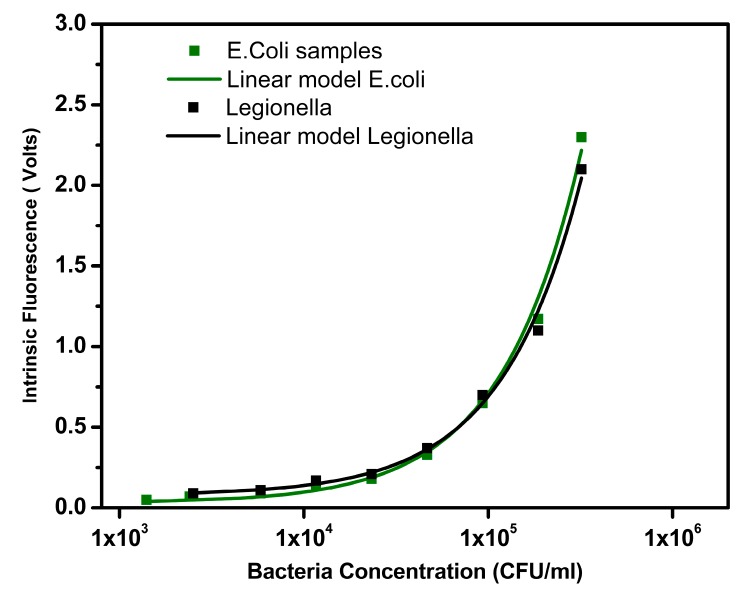
Linear model for intrinsic fluorescence signal on *E. coli* and *Legionella*.

To create the linear fitting model, the first two points where the sensor was saturated were removed from the graph; otherwise, the model would not be accurate in the operational range of the sensor. As we can see in Figure 9, both (*E. coli* and *Legionella*) datasets are linear: for the *E. coli* R^2^ = 0.99, with the fitting equation of y = 0.028 + 6.83^−6^x; for the *Legionella* R^2^ = 0.99, with the fitting equation of y = 0.076 + 6.1^−6^x. This shows that even in the prototype, measurements of the concentration show a linear relationship with tryptophan concentration.

#### 3.2.2. Recovery Tests

To test the calibration curves obtained in the previous subchapter, additional recovery tests were performed with spiked samples with known concentrations of *E. coli* and *Legionella*. For that, 3 samples of each bacteria were used (each test was repeated 3 times, and the presented values are the average), and a comparison between the concentration obtained from the calibration curve and the real concentration was performed. The recovery percentage was calculated through the quotient between the predict concentration by the calibration curve and the real concentration. For both bacteria, a recovery percentage between 86% and 104% (Table 2) was observed, which proves the reproducibility of the method.

### 3.3. Online Measurements

Online tests were performed with continuous flow going through the device. To replicating a contamination event scenario in the laboratory with a continuous flow situation, two flasks were used: one with uncontaminated tap water and the other with tap water contamined with *E. coli*. The pump inlet was alternated between both flasks to represent contamination events. The MATLAB Interface was built to have a minimum threshold that should be crossed for the event to be considered a contamination. This threshold implementation allows users to select a minimum value of bacteria to detect in water. Also, another function was implemented which allows the user to select how much time over the threshold the signal needs to be maintained to be considered a contamination event. This is manly to avoid random spikes, for example due to cavitation inside the device.

**Figure 10:**
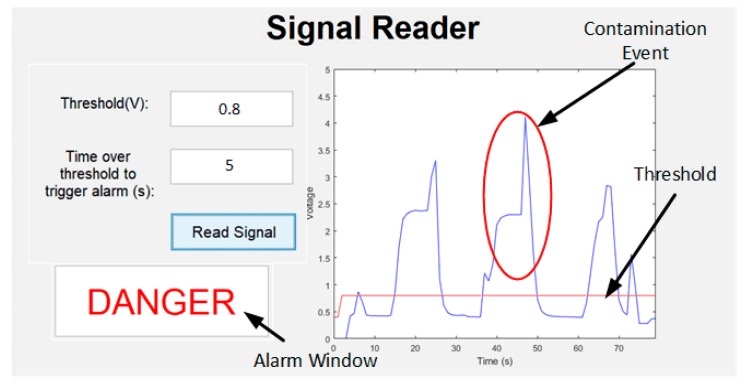
MATLAB interface operating, where is possible to see three detected contamination situations.

In Figure 10, it is possible to see 3 contamination spikes which the system was able to detect instantaneously. As an example, the threshold was set to 0.8 V, which corresponds to an *E. coli* concentration of approximately 10^5^ CFU/mL; the time for it to be considered a contamination was set a 5 s, which corresponds to 5 samples over the threshold limit. The calibration curve of the device was measured, and a similar limit of detection of 1.4 × 10^3^ CFU/mL was found with the offline and online tests.

## 4. Discussion

The spectrometry analysis shows a shift on the Trypthopan signal at a lower emission wavelength. This emission peak is mainly dependent on the solvent polarity and folded proteins in which the aminoacid is contained [29]. The solvent polarity can only cause a shift to lower wavelengths if the solvent has an high hydrophobicity, which is not the case; this means that the only possibility for the shift found on the on the *E. coli* spectroscopy can be explained by the tryptophan integration in *E. coli* proteins [20]. The Raman Scattering effect was within the expected values for water (i.e., Ex/Em = 280/310 nm), and is associated with the excitation light causing vibrational changes (inelastic scattering) on water molecules resulting in light reemission at higher wavelengths. As mentioned, this light can be blocked in the prototype by a filter. Nevertheless, the signal was able to distinguish this from the background noise, and the performed analysis supported the possibility of detecting this aminoacid within pathogen cells.

Offline and Online tests have proven that, even using low-power instrumentation, the device was able to detect the *E. coli* and *Legionella*. By performing the dilution series, it was possible to obtaining a correlation between the intrinsic Fluorescence signal and bacteria concentration. The response time for the system was in the milliseconds range, which outperforms any other method for detecting pathogens. The sensitivity obtained by the sensor was approximately 1.4 × 10^3^ CFU/mL, which was obtained using two low power UV-LEDs. This sensitivity can be drastically increased by using more powerful LEDs, for example SMD Deep UV LEDs, which have higher output power than traditional UV emitters [30]. Also, there is the possibility of coating the collection tube and analysis chamber with an UV reflective material such as aluminum [31] which causes multiple light reflections and increases the number of photon reaching the target bacteria’s and the detector, resulting in a higher signal. 

To completely reduce all external noise, an optimization of individual components for targeting unique fluorophore should be done. This optimization consists of fabricating a high specific transmission emission and excitation filter [32], which only allows the target wavelength to be transmitted.

## 5. Conclusions

Increased access to drinking water from pipelines has unquestionably increased the demand for water monitoring sensors. For an efficient approach, there is a necessity to implement an Internal Networking Monitoring strategy which requires a sensor with both NRT and low-cost operating capabilities. The preliminary Study on Intrinsic Fluorescence techniques show a huge potential for pathogen detection in drinking water. The presented instrumentation was able to achieve a linear range from 7 × 10^5^ CFU/mL to 1 × 10^4^ CFU/mL (*E. coli*), with a detection limit of 1.4 × 10^3^ CFU/mL. Theoretically, it is also possible for this method to detect other types of pathogens, such as viruses; this should be tested when the instrumentation’s efficiency has been maximized. Nevertheless, the simplicity behind the targeted Intrinsic Fluorescence technique makes the production of sensors based on this technique economically viable, and at the same time, perfectly matches the NRT requirements for Internal Networking Monitoring. To develop this system into a viable commercial product with practical value, further optimization and tests should be performed to decrease the limit of detection and prove a wider pathogenic detection range.

## Figures and Tables

**Table 1:** Basic Intrinsic Fluorescence characteristics of the essential amino acids [18,19].

Essential Amino Acids	Quantum Yield	Excitation/Emission * (nm)	Stoke Shift (nm)
Tryptophan	20%	280/350	70
Tyrosine	14%	274/300	26
Phenylalanine	4%	257/280	23

* Intrinsic fluorescence may change depending on the protein integration and solvent polarity [20].

**Table 2:** Value of the recovery performed recovery tests.

Pathogens	Spiked Sample (CFU/mL)	Recovery Percentage (%)
*E. coli*	7.1 × 10^5^	103.4
	8.6 × 10^4^	100.8
	9.0 × 10^3^	91.0
*Legionella*	6.9 × 10^5^	92.2
	8.2 × 10^4^	91.0
	9.5 × 10^3^	86.6
